# A case–control evaluation of pulmonary and extrapulmonary findings of incidental asymptomatic COVID-19 infection on FDG PET-CT

**DOI:** 10.1259/bjr.20211079

**Published:** 2021-04-29

**Authors:** Manil Subesinghe, Shaheel Bhuva, Joel T Dunn, Alexander Hammers, Gary J Cook, Sally F Barrington, Barbara M Fischer

**Affiliations:** 1 King’s College London & Guy’s and St. Thomas’ PET Centre, London, UK; 2 Department of Cancer Imaging, School of Biomedical Engineering and Imaging Sciences, King’s College London, London, UK

## Abstract

**Objectives:**

To describe the findings of incidental asymptomatic COVID-19 infection on FDG PET-CT using a case–control design.

**Methods:**

Incidental pulmonary findings suspicious of asymptomatic COVID-19 infection on FDG PET-CT were classified as a *confirmed* (positive RT-PCR test) or *suspected* case (no/negative RT-PCR test). *Control* cases were identified using a 4:1 control:case ratio. Pulmonary findings were re-categorised by two reporters using the BSTI classification. SUV metrics in ground glass opacification (GGO)/consolidation (where present), background lung, intrathoracic nodes, liver, spleen and bone marrow were measured.

**Results:**

7/9 *confirmed* and 11/15 *suspected* cases (COVID-19 group) were re-categorised as BSTI 1 (classic/probable COVID-19) or BSTI 2 (indeterminate COVID-19); 0/96 *control* cases were categorised as BSTI 1. Agreement between two reporters using the BSTI classification was almost perfect (weighted κ = 0.94). SUV_max_ GGO/consolidation (5.1 *vs* 2.2; *p* < 0.0001) and target-to-background ratio, normalised to liver SUV_mean_ (2.4 *vs* 1.0; *p* < 0.0001) were higher in the BSTI 1 & 2 group *vs* BSTI 3 (non-COVID-19) cases. SUV_max_ GGO/consolidation discriminated between the BSTI 1 & 2 group *vs* BSTI 3 (non-COVID-19) cases with high accuracy (AUC = 0.93). SUV metrics were higher (*p* < 0.05) in the COVID-19 group *vs control* cases in the lungs, intrathoracic nodes and spleen.

**Conclusion:**

Asymptomatic COVID-19 infection on FDG PET-CT is characterised by bilateral areas of FDG avid (intensity > x2 liver SUV_mean_) GGO/consolidation and can be identified with high interobserver agreement using the BSTI classification. There is generalised background inflammation within the lungs, intrathoracic nodes and spleen.

**Advances in knowledge:**

Incidental asymptomatic COVID-19 infection on FDG PET-CT, characterised by bilateral areas of ground glass opacification and consolidation, can be identified with high reproducibility using the BSTI classification. The intensity of associated FDG uptake (>x2 liver SUV_mean_) provides high discriminative ability in differentiating such cases from pulmonary findings in a non-COVID-19 pattern. Asymptomatic COVID-19 infection causes a generalised background inflammation within the mid-lower zones of the lungs, hilar and central mediastinal nodal stations, and spleen on FDG PET-CT.

## Introduction

The coronavirus disease 2019 (COVID-19) pandemic has created the biggest global health crisis in generations. The spread of infection has been difficult to control due to asymptomatic infection, estimated to account for over 50% of all transmissions.^1^ Nasopharyngeal swab reverse transcriptase polymerase chain reaction (RT-PCR) is considered the gold standard for diagnosing COVID-19 infection.^2,3^ The sensitivity of RT-PCR in symptomatic patients ranges between 82 and 97%^4^ but detection rates are lower in asymptomatic individuals.^5,6^

During the early stages of the pandemic in 2020, numerous studies described computed tomography (CT) features characteristic of COVID-19 infection with some suggesting sufficient diagnostic accuracy of CT in the absence of RT-PCR testing^7,8^ ; significant selection bias and several confounding factors have since undermined such conclusions.^9^ International guidelines and a recent umbrella review recommend CT as a problem-solving tool to identify complications of COVID-19 infection or when an alternative diagnosis is suspected in symptomatic individuals.^10,11^ The British Society of Thoracic Imaging (BSTI) published criteria for the diagnosis of COVID-19 infection on CT, based on the presence and distribution of ground glass opacification (GGO), consolidation and varied patterns of organising pneumonia (OP).^12^ Asymptomatic individuals can have normal lungs on CT or alternatively demonstrate radiological features compatible with COVID-19 infection.^13–15^

Several case reports/series of asymptomatic COVID-19 infection on 2-deoxy-2-[^18^F]fluoro-D-glucose (FDG) positron emission tomography (PET)-CT report metabolically active findings mainly confined to the lungs and mediastinal lymph nodes^16–27^ ; most studies have been purely descriptive, however. A few studies reporting increased FDG uptake in extrathoracic nodes, spleen, and bone marrow, suggest that FDG PET-CT can demonstrate the immune response to viral infections.^25–27^ Identifying incidental COVID-19 infection can alter patients’ immediate management and reduce the risk of transmission to others and is of particular importance to cancer patients who are at increased risk from COVID-19 infection.^28,29^

## Objective

Our hypothesis is that asymptomatic COVID-19 infection on FDG PET-CT imaging manifests as areas of FDG avid GGO/consolidation on the background of generalised inflammation in the lungs and other extrapulmonary locations. We will assess whether pulmonary and extrapulmonary findings on FDG PET-CT in patients with suspected asymptomatic COVID-19 infection scanned during the ‘first wave’ of UK pandemic, are significantly different to those in a control group scanned prior to the pandemic, matched for age, gender and scan indication. We will also determine the ability of FDG uptake in conjunction with pulmonary findings categorised using the BSTI classification to discriminate between COVID-19 and non-COVID-19 infection, whilst assessing the interobserver agreement between two reporters using the BSTI classification.

## Methods

### Case selection

Institutional review board approval was obtained for this retrospective non-interventional observational case–control study. Inclusion criteria were:

- FDG PET-CT examination performed between 23/03/2020 and 29/05/2020 during the ‘first wave’ of the UK pandemic.
- Absence of new continuous cough or high temperature, *i.e.,* asymptomatic.
- Expedited (via email notification) FDG PET-CT report due to incidental pulmonary findings suspicious for asymptomatic COVID-19 infection.

Referring clinicians either opted for confirmation of COVID-19 infection via RT-PCR hospital testing or recommendation for a period of self-isolation for 14 days as per UK government guidance due to a lack of community testing at the time.^30^ Patients with a positive RT-PCR test within 28 days of scanning were classified as a *confirmed* case. Patients with pulmonary findings suspicious of COVID-19 infection on FDG PET-CT but no or negative RT-PCR test within 28 days of scanning were classified as a *suspected* case. Information on RT-PCR testing and clinical follow-up was obtained from institutional electronic databases. Consecutive *control* cases matched for age, gender and scan indication without exclusion criteria were identified from spring 2019 ± 3 months, using a 4:1 control: case ratio.

### FDG PET-CT imaging review

FDG PET-CT examinations were performed using methodology aligned to EANM guidance and described previously.^31^ All examinations were anonymised (including date of examination) and analysed using Hybrid Viewer (Hermes Medical Solutions, Sweden). Independent blinded review of pulmonary findings was undertaken 6 months after the ‘first wave’ by board certified radiologists (S.B) and consultant radiologist (M.S) with 1 and 10 years of PET-CT reporting experience, respectively, and each with 12 years of diagnostic CT (including thoracic CT) reporting experience. Pulmonary findings were categorised using the BSTI classification^12^ ; classic/probable COVID-19 (BSTI 1), indeterminate COVID-19 (BSTI 2), non-COVID-19 (BSTI 3), and normal (BSTI 4). The normal category (BSTI 4) included findings considered within the spectrum of normality for PET-CT, *e.g.,* gravity-dependent GGO and basal linear atelectasis. Following independent review, examinations with disagreement in BSTI classification had consensus reads. For examinations with clinically significant pulmonary parenchymal findings, *i.e.,* BSTI 1–3, the highest maximum standardised uptake value (SUV_max_) in an area of GGO/consolidation was documented, enabling target-to-background ratio (TBR) calculation, normalised to the mean standardised uptake value (SUV_mean_) in the liver.

SUV metrics were derived from normal lung and from extrapulmonary sites (intrathoracic nodes, liver, spleen and bone marrow) by a consultant nuclear medicine physician (B.M.F) with 15 years of PET-CT reporting experience. Freehand regions of interest (ROIs) following the contours of the lungs but excluding subpleural regions and avoiding major vessels or parenchymal abnormalities were drawn in the upper (level of suprasternal notch), mid (1 cm below the carina) and lower zones (2.5 cm above the right hepatic dome) of both lungs to calculate SUV_mean_ of background lung. ROIs were drawn around the major intrathoracic nodal stations^32^ to calculate nodal SUV_max_; SUV_max_ was only measured if lymph nodes were visible on CT. Spherical volumes of interest (VOIs) were placed in the right lobe of the liver (6 cm diameter), spleen (3 cm diameter) and L4 vertebral body (2 cm diameter) as a representation of marrow uptake, to calculate SUV_mean_ in these VOIs.

### Statistical analysis

Interobserver agreement using the BSTI classification was assessed using the weighted κ method.^33^ Non-parametric tests were used to assess for group-wise (Kruskal-Wallis) and pairwise (Mann Whitney U) differences. The Benjamini-Hochberg method to estimate the false discovery rate (FDR) was used to correct for multiple comparisons; an FDR < 0.05 was considered significant. Receiver operating characteristic (ROC) curves were generated with an area under the curve (AUC) calculated for each ROC^34^ with the best thresholds for group discrimination defined using Youden’s method.^35^ All analyses were performed in R v. 4.0.0 with the base and stats packages while ROC analyses were performed using the pROC package. More detailed information can be found in the Supplementary Material 1

Supplementary Material 1.

## Results

732 FDG PET-CT examinations performed during spring 2020; 24 (3.3%) examinations had incidental pulmonary findings suspicious for asymptomatic COVID-19 infection. Nine patients had RT-PCR confirmation of COVID-19 infection (range 0–22 days from scanning), *i.e., confirmed* cases, and 15 remained *suspected* cases; these together comprised the COVID-19 group. Twelve out of 15 *suspected* cases self-isolated at home without access to RT-PCR testing in the community (Table 1, Supplementary Table 1). There were 96 matched *control* cases; 20 of which had visible areas of GGO/consolidation eligible for SUV_max_ and TBR analysis. A total of 120 anonymised examinations were independently reviewed and analysed (FDG injection: 329 ± 24 MBq (range 285–392 MBq), mean uptake time: 63 ± 4.5 min (range 56–78 min)).

Supplementary Table 1.

**Table 1.** Details of *confirmed* and *suspected* cases of COVID-19 infection

Case	Age (years)	Gender	Scan indication	SUV_max_ GGO/consolidation	BSTI classification	RT-PCR status (days after FDG PET-CT)	COVID-19 status	6-month imaging follow-up
1	59	Male	Head & Neck cancer	7.2	1	Negative (1 day)	Suspected	Resolution on 4 month f/u PET-CT.
2	65	Female	Melanoma	3.6	3	None	Suspected	Resolution on 2 month f/u CT thorax.
3	42	Female	Lymphoma	7.0	3	Positive (0 days)	Confirmed	Pulmonary fibrosis on 6 month f/u CT thorax.
4	72	Female	Head & Neck cancer	6.1	2	None	Suspected	Resolution on 2 month f/u CT thorax.
5	52	Male	Oesophageal cancer	8.7	1	Positive (22 days)	Confirmed	Resolution on 3 month f/u PET-CT.
6	86	Male	Melanoma	5.6	2	None	Suspected	No further imaging.
7	76	Male	Cardiac infection	4.1	1	Positive (3 days)	Confirmed	No further imaging.
8	63	Female	Myeloma	3.2	2	None	Suspected	Resolution on 5 month f/u chest radiograph.
9	66	Male	Lymphoma	6.7	1	None	Suspected	Resolution on 4 month f/u CT thorax.
10	51	Male	Lymphoma	3.5	3	None	Suspected	No further imaging.
11	69	Male	Melanoma	3.9	1	None	Suspected	Resolution on 3 month f/u PET-CT.
12	54	Male	Pancreatic cancer	7.8	1	Positive (7 days)	Confirmed	Resolution on 4 month f/u CT thorax.
13	49	Female	Lymphoma	3.8	2	None	Suspected	Resolution on 1 month f/u PET-CT.
14	66	Female	Endometrial cancer	2.0	1	Negative (1 day)	Suspected	Resolution on 2 month f/u PET-CT.
15	64	Female	Lung cancer	2.1	1	None	Suspected	Resolution on 2 month f/u PET-CT.
16	51	Female	Lymphoma	0.7	3	None	Suspected	No further imaging.
17	49	Female	Oesophageal cancer	2.3	3	None	Suspected	No further imaging.
18	39	Female	Unknown malignancy	4.0	2	Negative (1 day)	Suspected	No further imaging.
19	33	Female	Cutaneous lymphoma	1.4	3	Positive (16 days)	Confirmed	Resolution on 3 month f/u chest radiograph.
20	87	Male	Melanoma	6.1	1	None	Suspected	Resolution on 5 month f/u PET-CT.
21	22	Male	Lymphoma	5.9	1	Positive (0 days)	Confirmed	Resolution on 3 month f/u PET-CT.
22	60	Male	Lymphoma	5.0	1	Positive (9 days)	Confirmed	Pulmonary fibrosis on 3 month f/u CT thorax.
23	54	Female	Lymphoma	7.1	1	Positive (0 days)	Confirmed	Partial resolution on 2 month f/u PET-CT.
24	56	Female	Lymphoma	6.0	1	Positive (0 days)	Confirmed	Resolution on 5 month f/u PET-CT.

BSTI, British Society of Thoracic Imaging; GGO, ground glass opacification; RT-PCR, reverse transcriptase-polymerase chain reaction; SUV_max_, maximum standardised uptake value; f/u, follow up.

BSTI 1 = classic/probable COVID-19; BSTI 2 = indeterminate COVID-19; BSTI 3 = non-COVID-19;

### BSTI classification

**Figure 1.**
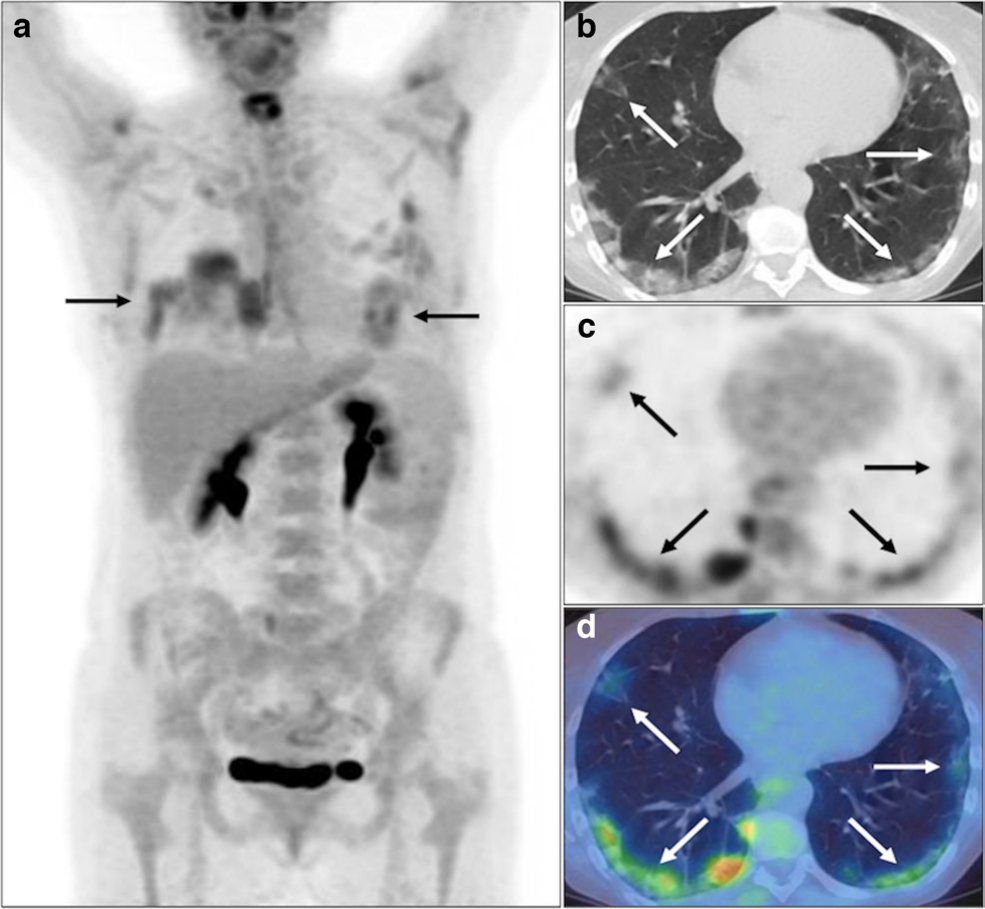
Bilateral FDG avid subpleural GGO and consolidation with perilobular opacity, *i.e.,* OP pattern (solid black and white arrows), and a mid-lower zone predominance, *e.g.,* right lower lobe (SUV_max_ 6.0) on PET MIP (**A**), axial CT, PET and fused PET-CT (**B-D**). BSTI 1 with positive RT-PCR (*confirmed* case).

**Figure 2.**
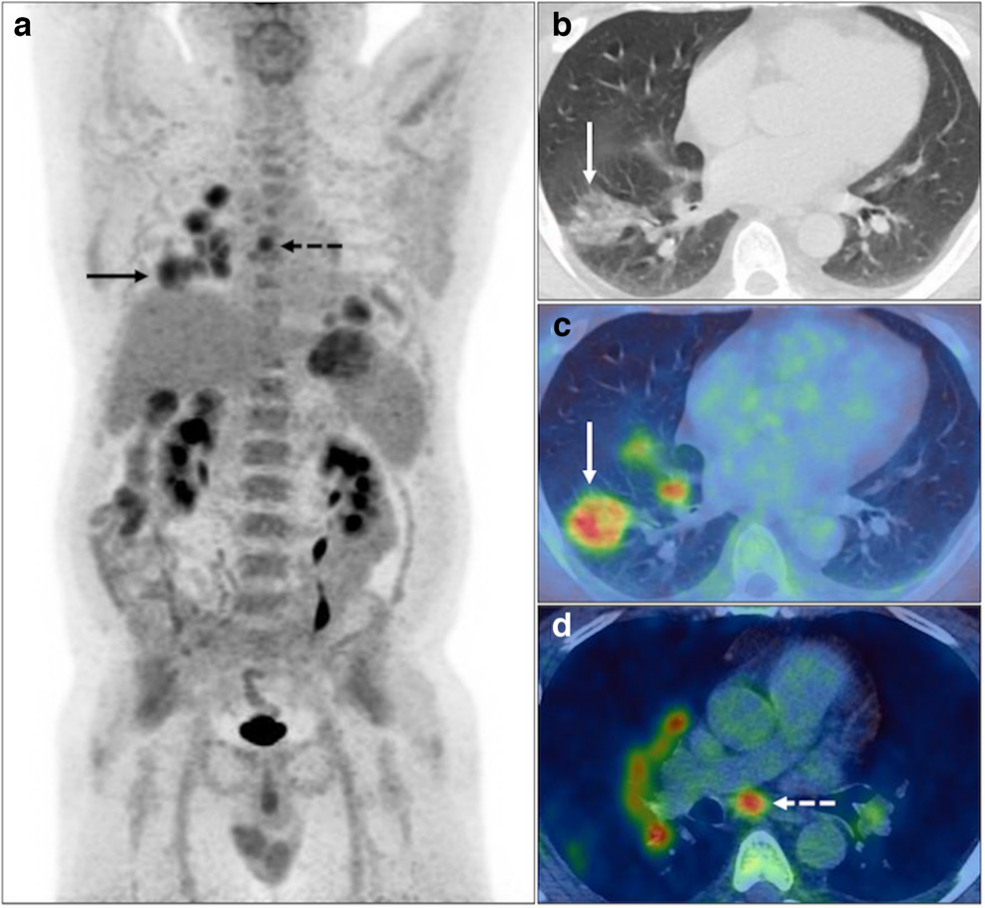
Bilateral (right>left) FDG avid (SUV_max_ 8.7) nodular consolidation including foci of central GGO with surrounding circumferential consolidation, *i.e*. ‘reverse-halo’ sign, in the right lower lobe (solid black and white arrows), with reactive non-enlarged FDG avid (SUV_max_ 6.5) subcarinal lymph node (dashed black and white arrows) on PET MIP (**A**), axial CT and fused PET-CT (**B-D**). BSTI 1 with positive RT-PCR (*confirmed* case).

**Figure 3.**
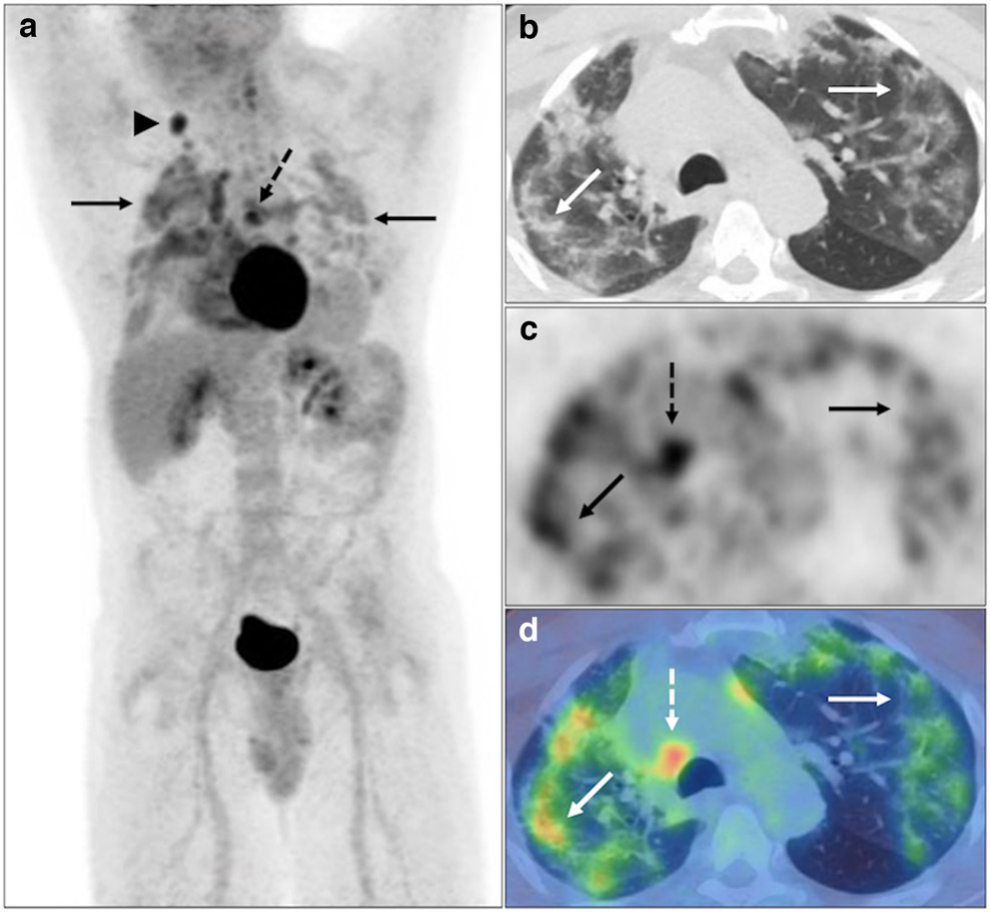
Widespread FDG avid central and subpleural GGO and consolidation with perilobular opacity (solid black and white arrows), *e.g.,* right upper lobe (SUV_max_ 6.7) with reactive borderline normal sized right lower paratracheal (dashed black and white arrows) and right supraclavicular fossa nodes (black arrowhead) on PET MIP (**A**), axial CT, PET and fused PET-CT (**B-D**). BSTI 1 without RT-PCR testing (*suspected* case).

**Figure 4.**
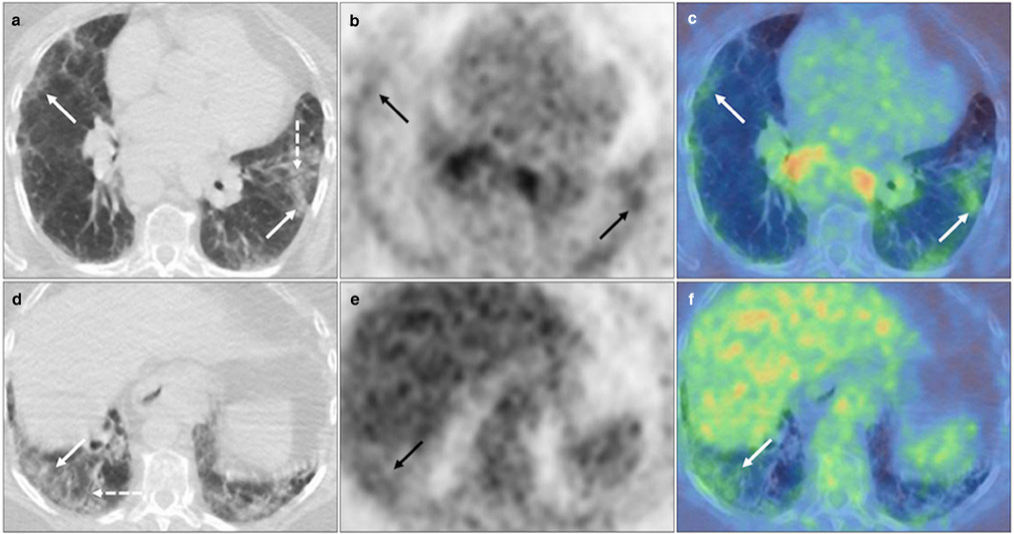
Bilateral FDG avid subpleural GGO with lower zone predominance (solid black and white arrows), *e.g.,* left lower lobe (SUV_max_ 3.4) and subtle co-existent tractional airways dilatation (dashed white arrows) on axial CT, PET and fused PET-CT (A-C, 5 F). BSTI 2 but appearances represented pulmonary fibrosis secondary to sarcoidosis in a *control* case.

**Figure 5.**
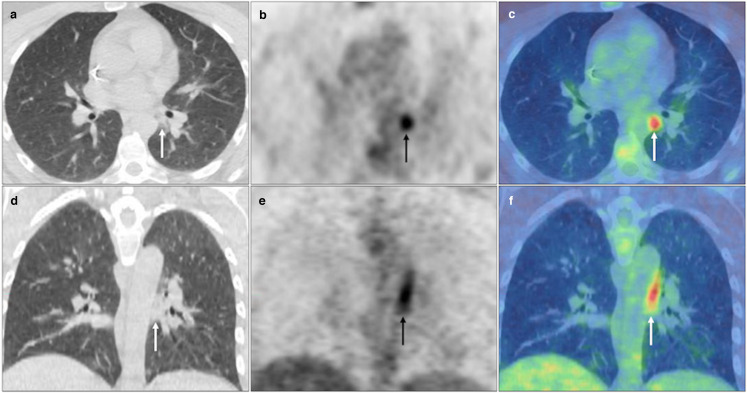
Unilateral FDG avid (SUV_max_ 7.0) vertically orientated consolidation posterior to the left lower lobe bronchus (solid black and white arrows) on axial CT, PET and fused PET-CT (**A-C, D-F**). BSTI 3 with positive RT-PCR (*confirmed* case).

**Figure 6.**
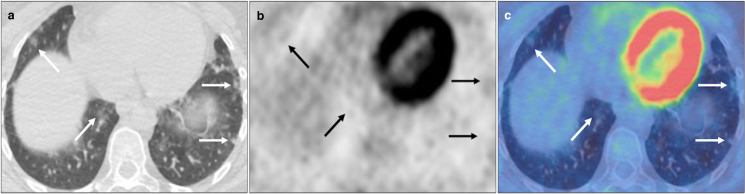
Small bilateral subpleural nodular foci of GGO demonstrating low-grade FDG uptake (solid black and white arrows), *e.g*. left lower lobe (SUV_max_ 1.4) on axial CT, PET and fused PET-CT (**A-C**). BSTI 3 with positive RT-PCR (*confirmed* case).

**Figure 7.**
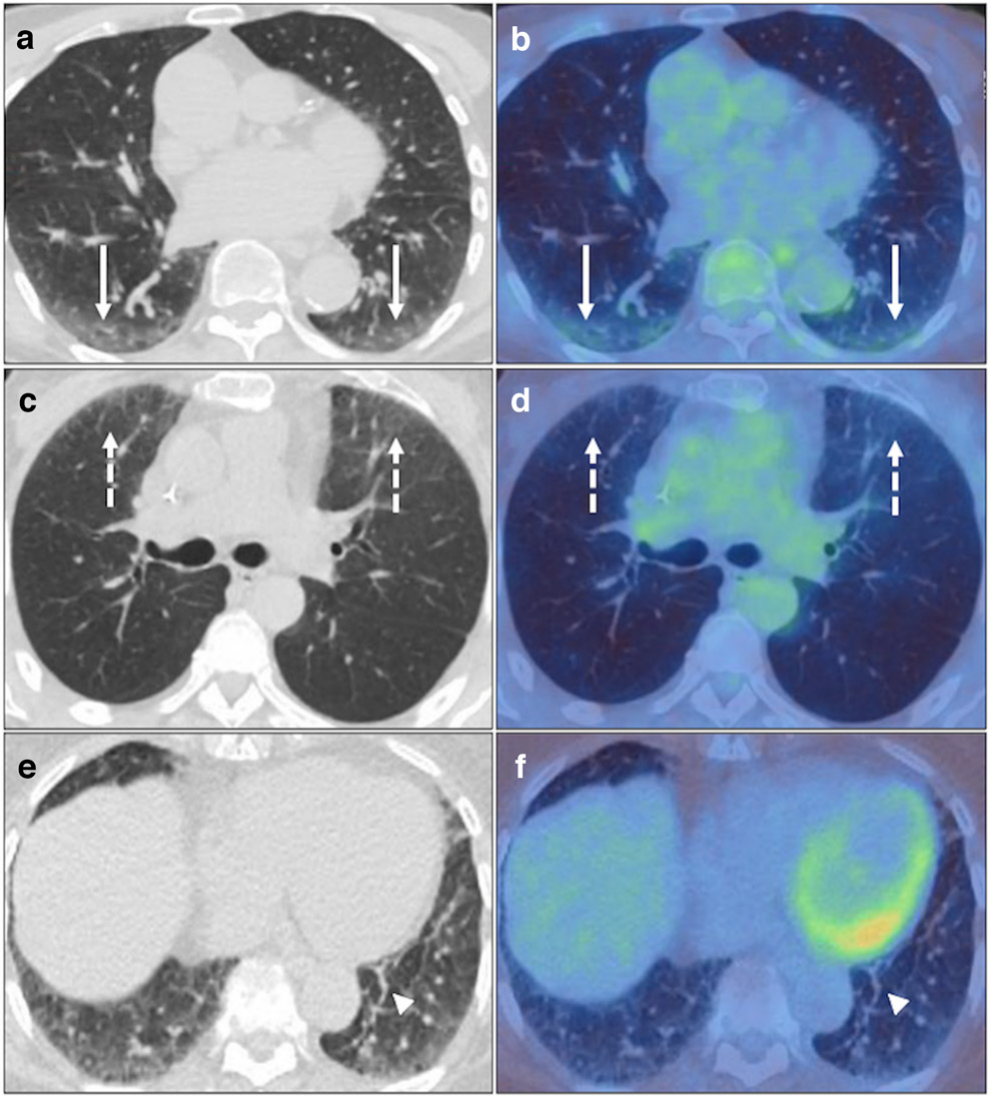
Frequently observed clinically insignificant pulmonary findings on non-breath hold FDG PET-CT considered within the spectrum of normality for PET-CT, i.e. BSTI 4, including bilateral-dependent GGO with low-grade FDG uptake in the posterior lower lobes (solid white arrows) with supine scanning on axial CT and fused PET-CT (A&B), anterior upper lobes (dashed white arrows) with prone scanning on axial CT and fused PET-CT (C&D), and linear basal atelectasis without FDG uptake (white arrowheads) on axial CT and fused PET-CT (E&F).

7/9 *confirmed* and 11/15 *suspected* cases, were categorised as BSTI 1 or 2 (Figures 1–3) , 0/96 *control* cases were categorised as BSTI 1, and only two *control* cases categorised as BSTI 2 (Figure 4). 2/9 *confirmed* cases (Figures 5 and 6) and 4/15 *suspected* cases were categorised as BSTI 3, whilst the remaining *control* cases were categorised as BSTI 3 (18/96) or BSTI 4 (76/96) (Figure 7). The BSTI classification had a sensitivity of 75% and specificity of 97.9% for the detection of COVID-19 infection on the CT component of FDG PET-CT, assuming that only BSTI 1 and 2 appearances represent COVID-19 infection.

There was almost perfect agreement (weighted κ = 0.94) between the two reporters using the BSTI classification across all four categories with an overall agreement of 94% (113/120). Excluding BSTI 4, which had 100% agreement (76/76), there remained almost perfect agreement for the BSTI 1–3 categories (weighted κ = 0.83) with an overall agreement of 84% (37/44); cases with disagreement only differed by one category between reporters (Table 2).

**Table 2.** Categorisation of pulmonary findings using the BSTI classification between two reporters

		REPORTER 1				
		BSTI 1	BSTI 2	BSTI 3	BSTI 4	TOTAL
**REPORTER 2**	**BSTI 1**	**12**	2	0	0	14
	**BSTI 2**	1	**5**	2	0	8
	**BSTI 3**	0	2	**20**	0	22
	**BSTI 4**	0	0	0	**76**	76
	**TOTAL**	13	9	22	76	**120**

BSTI 1, classic/probable COVID-19; BSTI 3, non-COVID-19; BSTI, British Society of Thoracic Imaging; BSTI 2, indeterminate COVID-19; BSTI 4, normal.

### SUV_max_ and TBR GGO/consolidation

**Figure 8.**
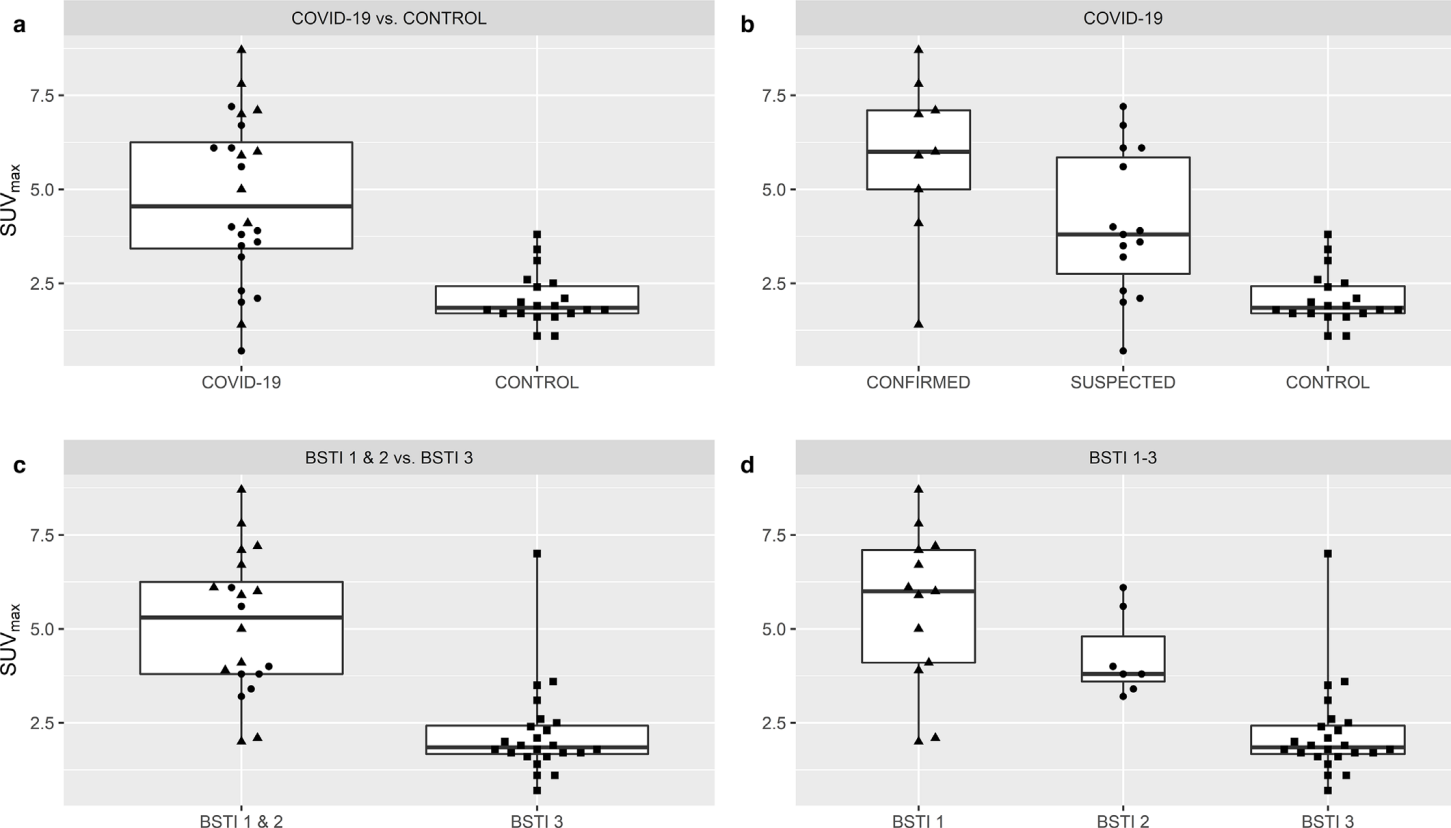
Scatter and box plots demonstrating differences in SUV_max_ GGO/consolidation across the COVID-19 (*confirmed vs suspected vs control*) and BSTI classifications (BST1 *vs* BSTI 2 *vs* BSTI 3) including aggregated groupings, COVID-19 and BSTI 1 & 2 (▲=CONFIRMED, ●=SUSPECTED, ■=CONTROL cases). Thick horizontal solid bar across the box shows the median, box height shows interquartile range (25-75th percentiles) and whiskers show minimum and maximum values.

**Figure 9.**
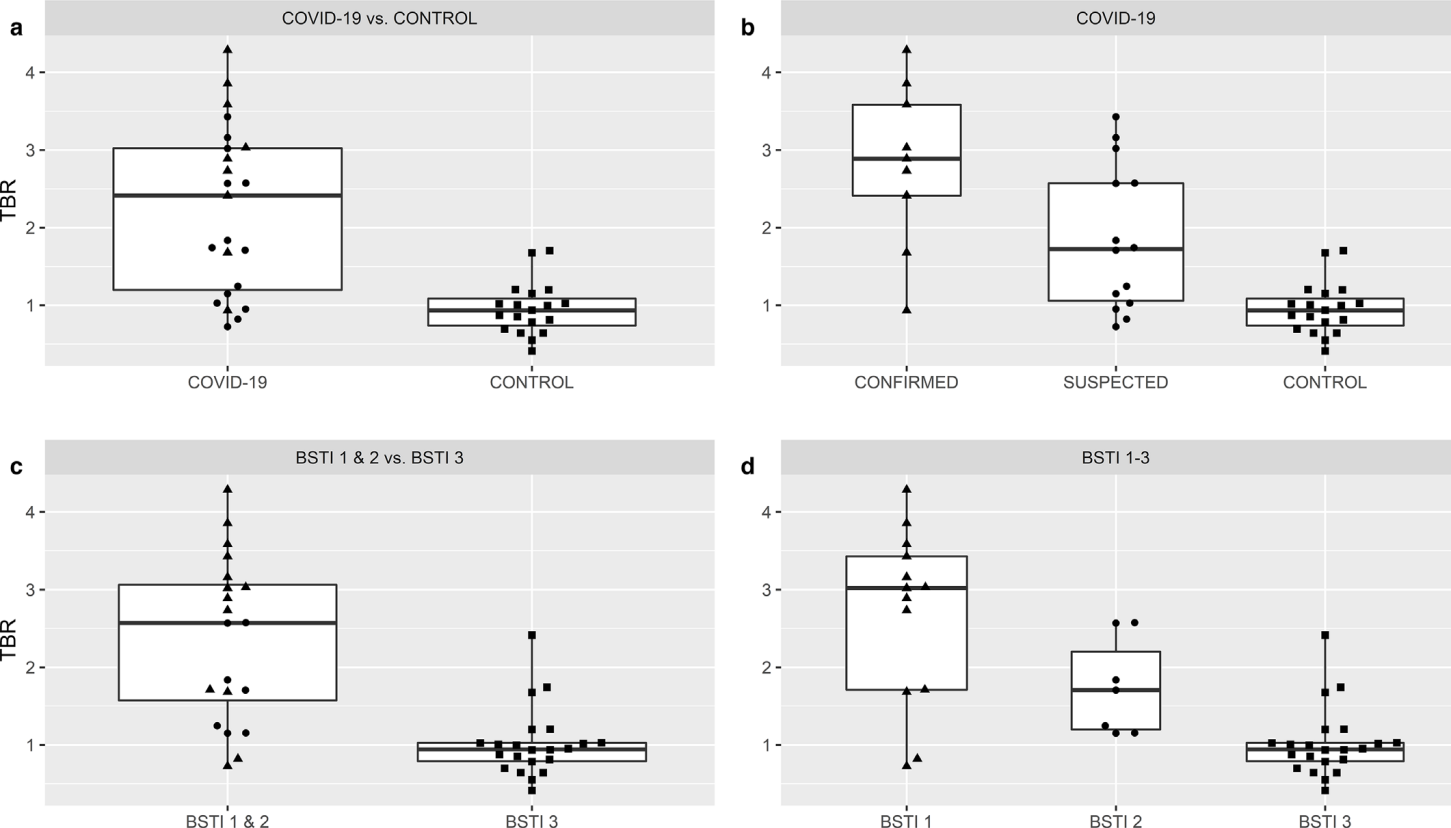
Scatter and box plots demonstrating differences in TBR GGO/consolidation across the COVID-19 (*confirmed vs suspected vs control*) and BSTI classifications (BST1 *vs* BSTI 2 *vs* BSTI 3) including aggregated groupings, COVID-19 and BSTI 1 & 2 (▲=CONFIRMED, ●=SUSPECTED, ■=CONTROL cases). Thick horizontal solid bar across the box shows the median, box height shows interquartile range (25-75th percentiles) and whiskers show minimum and maximum values.

There were highly significant group-wise differences (*p* < 0.0001) across both the COVID-19 and BSTI classifications. Pairwise comparisons across the COVID-19 classification revealed no difference in SUV_max_ (*p* = 0.056) or TBR (*p* = 0.066) GGO/consolidation between *confirmed vs suspected* cases after correction for multiple comparisons. SUV_max_ GGO/consolidation was, however, significantly higher in the COVID-19 group (*confirmed* and *suspected* cases) *vs control* cases (4.7 *vs* 2.1; *p* < 0.0001) as was TBR (2.2 *vs* 1.0; *p* < 0.0001), (Tables 3 and 4, Figures 8 and 9).

**Table 3.** Association of SUV_max_ GGO/consolidation by COVID-19 status and BSTI classification

GROUP	N	Minimum	Median	Maximum	Mean	SD
**CONFIRMED**	9	1.4	6	8.7	5.9	2.2
**SUSPECTED**	15	0.7	3.8	7.2	4.1	1.9
**CONTROL**	20	1.1	1.9	3.8	2.1	0.7
**COVID-19**	24	0.7	4.6	8.7	4.7	2.2
**BSTI 1**	13	2	6	8.7	5.6	2.1
**BSTI 2**	7	3.2	3.8	6.1	4.3	1.1
**BSTI 3**	24	0.7	1.9	7	2.2	1.2
**BSTI 1 & 2**	20	2	5.3	8.7	5.1	1.9
**GROUP COMPARISON**	**UNCORRECTED *P*-VALUE**				**FDR**	
**COVID CLASSIFICATION KRUSKAL-WALLIS**	<0.0001^*a*^				-	
**BSTI CLASSIFICATION KRUSKAL-WALLIS**	<0.00001^*a*^				-	
**PAIRWISE COMPARISON**						
**CONFIRMED *vs* SUSPECTED**	0.049^*a*^				0.056	
**CONFIRMED *vs* CONTROL**	0.00074^*a*^				0.0012^*a*^	
**SUSPECTED *vs* CONTROL**	0.00046^*a*^				0.00092^*a*^	
**COVID-19 *vs* CONTROL**	<0.0001^*a*^				0.00010^*a*^	
**BSTI 1 *vs* BSTI 2**	0.088				0.088	
**BSTI 1 *vs* BSTI 3**	<0.0001^*a*^				0.00010^*a*^	
**BSTI 2 *vs* BSTI 3**	0.00061^*a*^				0.0011^*a*^	
**BSTI 1 & 2 *vs* BSTI 3**	<0.00001^*a*^				<0.0001^*a*^	

BSTI, British Society of Thoracic Imaging; FDR, False Discovery Rate; GGO, ground glass opacification; N, number of cases; SD, standard deviation; SUVmax, maximum standardised uptake value.

BSTI 1 = classic/probable COVID-19; BSTI 2 = indeterminate COVID-19; BSTI 3 = non-COVID-19, COVID-19 group = confirmed and suspected cases (confirmed = pulmonary findings suspicious of COVID-19 infection on FDG PET-CT and a positive RT-PCR test within 28 days of scanning; suspected = pulmonary findings suspicious of COVID-19 infection on FDG PET-CT but no/negative RT-PCR test within 28 days)

a statistically significant

**Table 4.** Association of TBR GGO/consolidation grouped by COVID-19 status and BSTI

**GROUP**	**N**	**Minimum**	**Median**	**Maximum**	**Mean**	**SD**
**CONFIRMED**	9	0.9	2.9	1.7	2.8	1.1
**SUSPECTED^*b*^**	14	0.7	1.7	3.4	1.9	0.9
**CONTROL^*b*^**	19	0.4	0.9	1.7	1.0	0.3
**COVID-19**	23	0.7	2.4	4.3	2.2	1.1
**BSTI 1**	13	0.7	3.0	4.3	2.7	1.1
**BSTI 2**	7	1.2	1.7	2.6	1.8	0.6
**BSTI 3**	22	0.4	0.9	2.4	1.0	0.4
**BSTI 1 & 2**	20	0.7	2.6	4.3	2.4	1.1
		
**GROUP COMPARISON**	**UNCORRECTED *P*-VALUE**				**FDR**	
**COVID CLASSIFICATION KRUSKAL-WALLIS**	<0.0001^*a*^				-	
**BSTI CLASSIFICATION KRUSKAL-WALLIS**	<0.00001^*a*^				-	
		
**PAIRWISE COMPARISON**	**UNCORRECTED *P*-VALUE**				**FDR**	
**CONFIRMED *vs* SUSPECTED**	0.062				0.066	
**CONFIRMED *vs* CONTROL**	<0.0001^*a*^				0.00015^*a*^	
**SUSPECTED *vs* CONTROL**	0.0012^*a*^				0.0016^*a*^	
**COVID-19 *vs* CONTROL**	<0.0001^*a*^				<0.0001^*a*^	
**BSTI 1 *vs* BSTI 2**	0.056				0.064	
**BSTI 1 *vs* BSTI 3**	0.00011^*a*^				0.00025^*a*^	
**BSTI 2 *vs* BSTI 3**	0.00093^*a*^				0.0014^*a*^	
**BSTI 1 & 2 *vs* BSTI 3**	<0.00001^*a*^				<0.0001^*a*^	

BSTI, British Society of Thoracic Imaging; FDR, False Discovery Rate; GGO, ground glass opacification; N, number of cases; SD, standard deviation; SUVmax, maximum standardised uptake value.

BSTI 1 = classic/probable COVID-19; BSTI 2 = indeterminate COVID-19; BSTI 3 = non-COVID-19, COVID-19 group = confirmed and suspected cases (confirmed = pulmonary findings suspicious of COVID-19 infection on FDG PET-CT and a positive RT-PCR test within 28 days of scanning; suspected = pulmonary findings suspicious of COVID-19 infection on FDG PET-CT but no/negative RT-PCR test within 28 days)

a statistically significant

b Hepatic disease involvement precluded liver SUVmean measurement and resultant TBR calculation in a single suspected case and single control case;

Pairwise comparisons across the BSTI classification revealed no differences in SUV_max_ GGO/consolidation (*p* = 0.088) or TBR (*p* = 0.064) between BSTI 1 and 2 cases. SUV_max_ GGO/consolidation was however significantly higher in the BSTI 1 & 2 group *vs* BSTI 3 cases (5.1 *vs* 2.2; *p* < 0.0001) as was TBR (2.4 *vs* 1.0; *p* < 0.0001) (Tables 3 and 4, Figures 8 and 9).

### SUV_max_ GGO/consolidation ROC analysis

**Figure 10.**
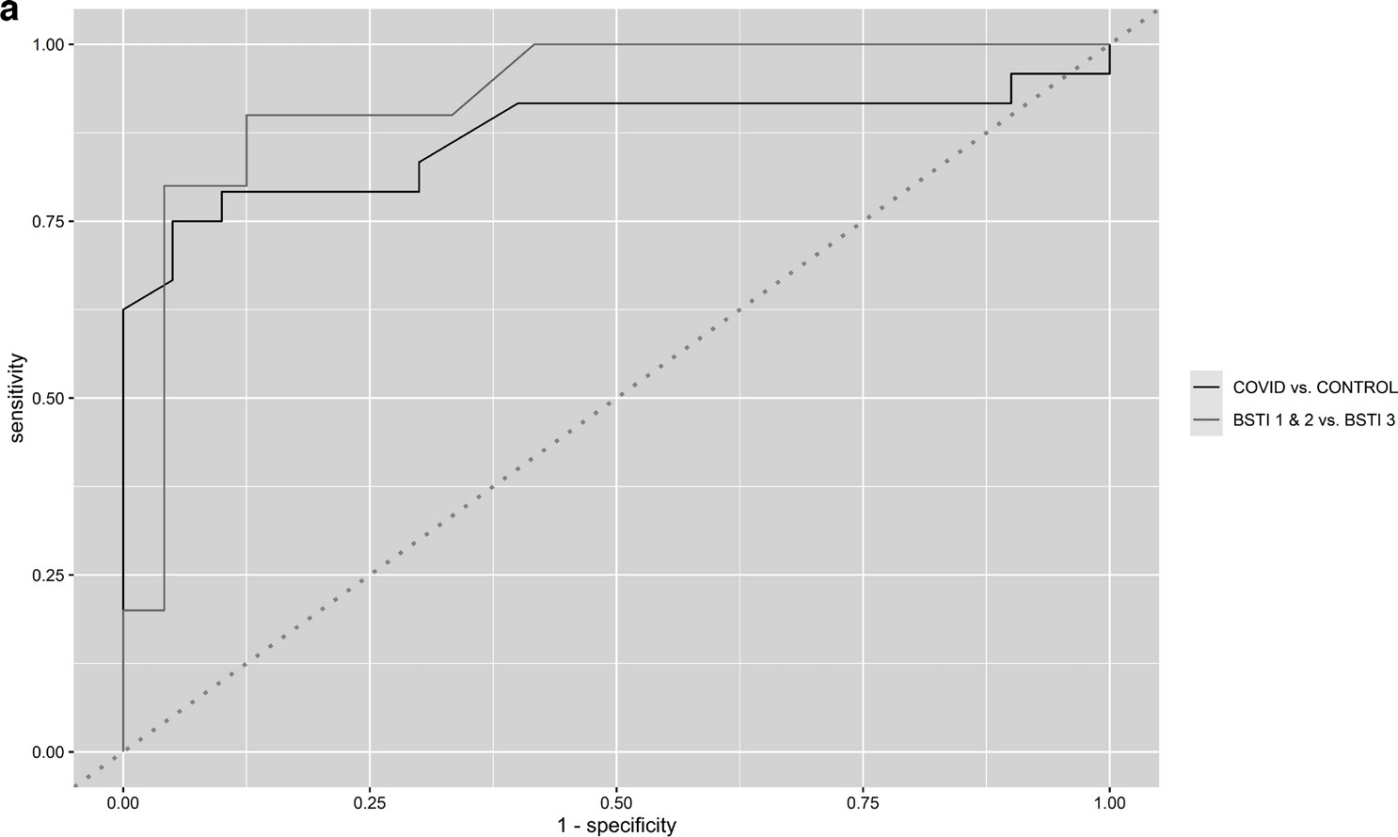
ROC curves determining the diagnostic performance of SUV_max_ GGO/consolidation on FDG PET-CT using the aggregated groupings COVID-19 group *vs control* cases and BSTI 1 & 2 group *vs* BSTI 3 cases.

ROC curves indicated excellent discrimination using SUV_max_ GGO/consolidation with an AUC of 0.93 (0.84–1.00) for differentiating between the BSTI 1 & 2 group and BSTI 3 cases and 0.87 (0.75–0.99) between the COVID-19 group and *control* cases (Figure 10). Using a SUV_max_ 3.15 cut-off, discrimination between the BSTI 1 & 2 group and BSTI 3 cases was achievable with a sensitivity of 0.90 and specificity of 0.88, whilst a SUV_max_ 3.45 cut-off enabled discrimination between the COVID-19 group and *control* cases with a sensitivity of 0.75 and specificity of 0.95.

### SUV metrics in pulmonary and extrapulmonary sites

There were significantly higher SUV metrics (*p* < 0.05) in the COVID-19 group *vs control* cases in 9/15 regions; 3/6 pulmonary regions (right mid zone, right lower zone, left lower zone), 5/6 nodal regions (bilateral hilar, bilateral paratracheal and subcarinal mediastinal nodal stations) and in the spleen. There was no significant difference in SUV_mean_ in the liver or bone marrow (Supplementary Table 2, Supplementary Figure 1).

Supplementary Table 2.

Supplementary Figure 1.

## Discussion

3.3% (24/732) of FDG PET-CT examinations performed during spring 2020 had incidental pulmonary findings suspicious of asymptomatic COVID-19 infection, which is within the quoted incidence range (2.1–16.2%) from a systematic review of 11 studies.^36^ Our incidence is lower than reported in a study from a similar sized London institution (9.4%),^19^ but this may be related to potential false-positive observations secondary to unilateral rather bilateral pulmonary findings, *i.e.,* indeterminate for COVID-19 (BSTI 2) coupled with most of their cases with thoracic findings on PET-CT, either negative (4/15) or without RT-PCR confirmation (10/15).

Blinded consensus pulmonary analysis performed 6 months later, with a greater experience of reporting COVID-19 infection on FDG PET-CT, categorised 18/24 of the *confirmed* and *suspected* cases as either BSTI 1 (classic/probable COVID-19) or BSTI 2 (indeterminate for COVID-19), whilst none of the control cases from 2019 were categorised as BSTI 1. This confirms that the pattern of FDG avid pulmonary parenchymal changes observed in spring 2020 (Figures 1–3) was a novel phenomenon not experienced before, and also demonstrates the high specificity (97.9%) achieved through using the BSTI classification.

Several studies report an increased incidence of pulmonary findings suspicious for COVID-19 infection during the ‘first wave’ compared to control cases^20–22,24^ similar to ours, except that patterns compatible with COVID-19 interstitial pneumonia were observed in their control cohorts; this is likely due to the presence of COVID-19 mimics on FDG PET-CT, *e.g.,* influenza pneumonia or OP related to connective tissue disease or drug toxicity. However, most studies did not use a standardised CT grading system for categorising pulmonary changes, likely reducing specificity. Maurea et al^20^ using the COVID-19 Reporting and Data System (CORADS)^37^ reported 14/335 (4%) control cases with ‘abnormal PET-CT findings suspicious for COVID-19 infection’. However, 9/14 (64%) were classified as CO-RADS 2 (CT abnormalities consistent infection other than COVID-19) or CO-RADS 3 (uncertain CT findings for COVID-19) suggesting an overestimation of PET-CT findings suspicious for COVID-19 infection in their control population.

Our study, the first to formally assess agreement between two reporters using the BSTI classification on FDG PET-CT, demonstrated almost perfect agreement (weighted κ = 0.94), even when BSTI 4 cases were excluded from the analysis (weighted κ = 0.83). Inui et al,^38^ compared different CT grading systems for COVID-19 infection, and showed that all had reasonable diagnostic performance (0.80–0.84), albeit with lower interobserver agreement (Cohen κ = 0.61–0.63) than ours, but that CO-RADS and BSTI outperformed the other two classifications. Our higher interobserver agreement may be augmented by amalgamation of the ‘classic’ and ‘probable’ COVID-19 categories to represent BSTI 1 as per the published COVID-19 CT reporting proforma^12^ rather than interpretate them as two separate categories. The inadequacies of the low-dose non-breath hold CT component of a PET-CT examination, requiring a more pragmatic approach to assessing the lungs, *i.e.,* forgoing subtleties, also likely contributed to more consistent and reproducible observations.

FDG uptake in areas of GGO/consolidation was significantly higher in the COVID-19 group *vs control* cases (SUV_max_ 4.7 *vs* 2.1) and BSTI 1 & 2 group *vs* BSTI 3 cases (SUV_max_ 5.1 *vs* 2.2); similar values have been reported in an early systematic review of incidental COVID-19 infection on FDG PET-CT (mean SUV_max_ 4.9),^39^ and Italian multicentre study (mean SUV_max_ 4.1).^24^ TBR analysis demonstrated that the intensity of FDG uptake in GGO/consolidation was >x2 liver SUV_mean_ in the COVID-19 and BSTI 1 & 2 groups, and lower and comparable to liver SUV_mean_ for *control* and BSTI 3 cases. These findings confirm that a distinctive feature of COVID-19 infection is the association of high FDG uptake with areas of GGO/consolidation, related to multinucleated giant cells and focal clusters of lymphomonocytic infiltration in the context of diffuse alveolar damage, demonstrable even in early COVID-19 infection.^40,41^

The discriminative ability of SUV_max_ in areas of GGO/consolidation to differentiate between the BSTI 1 & 2 group and BSTI 3 cases was high; using a SUV_max_ 3.15 cut-off, discrimination between BSTI 1 & 2 group and BSTI 3 cases was achievable with high sensitivity and specificity. From a clinical viewpoint, pulmonary findings compatible with classic/probable COVID-19 (BSTI 1), *e.g.,* bibasal peripheral GGO/consolidation with ‘reverse-halo’ or perilobular opacity, i.e., OP pattern, or indeterminate COVID-19 (BSTI 2), *e.g.,* unilateral, non-peripheral GGO/consolidation, are likely to have higher levels of associated FDG uptake (SUV_max_ >3.15) in comparison with GGO/consolidation in a non-COVID-19 (BSTI 3) pattern.

Our study reaffirms that the low-dose non-breath hold CT component of the study can enable diagnosis despite not being of ‘diagnostic quality’ and is not solely for the purposes of attenuation correction and localisation. The absence of a full inspiratory effort and breathing artefact during scanning can limit accuracy, however. Difficulty in detection/characterisation of smaller lesions particularly towards the lung bases limits sensitivity, whilst an increased incidence of dependent GGO alongside areas of basal atelectasis, can be potentially misinterpreted as significant pathology, reducing specificity. Unrelated pulmonary pathologies, *e.g.,* other viral pneumonias, OP secondary to connective tissue disease or drug toxicity or active pulmonary fibrosis can have similar appearances to COVID-19 infection, and will reduce specificity, although in our study this was only encountered in 2/96 control cases (Figure 4).

We found significantly higher SUV metrics in the COVID-19 group *vs control* cases in the mid-lower lung zones, both hilar and central mediastinal nodal stations, and spleen, suggesting the presence of generalised background inflammation. Lower zone predominant background pulmonary inflammation correlates with the tendency of COVID-19 infection to present with bilateral abnormalities affecting both lower lobes.^42,43^ FDG avid intrathoracic and supraclavicular nodes with COVID-19 infection have been reported in several studies with varied frequency, with or without CT enlargement,^20,22–25^ whilst only one study has reported increased splenic uptake (5/13 patients) and increased bone marrow uptake^25^ ; extrapulmonary abnormalities involving the salivary glands^19^ and gastro-intestinal tract^18^ were not routinely assessed for during our study. The presence of generalised systemic inflammation has been confirmed in small cohorts of patients recovering from COVID-19 infection (lungs, mediastinal nodes, spleen, liver, large vessels),^44,45^ as well as in patients with post-COVID syndrome^46^ in conjunction with findings of brain hypometabolism.^47,48^

The major limitation to our study, common to many, is the absence of RT-PCR testing for all FDG PET-CT examinations during spring 2020, due to a lack of testing capacity.^30^ Patients with COVID-19 infection but without pulmonary findings suspicious of infection will have been missed using our methodology, which will undoubtedly affect the sensitivity and specificity estimate of the BSTI classification on FDG PET-CT. In addition, this limitation also brings into question the classification of *suspected* cases which had either no (12/15) or a negative (3/15) RT-PCR test despite the presence of pulmonary findings suspicious of COVID-19 infection on FDG PET-CT (Supplementary Table 1). However, SUV_max_ GGO/consolidation and TBR analysis demonstrated that *confirmed* and *suspected* cases were similar to each other but were individually as well as in combination (COVID-19 group), distinct from *control* cases, supporting our methodology of combining these cases for analysis (Tables 3 and 4, Figures 8 and 9). In addition, the sensitivity of the gold standard nasopharyngeal RT-PCR in symptomatic individuals is not 100%^4^ and is lower in asymptomatic individuals, i.e., higher false-negative rates.^5,6^ Sensitivity can be improved through repeat RT-PCR testing^49^ or with bronchoalveolar lavage; a study of 46 patients reported 18 patients (39%) had a positive bronchoalveolar lavage RT-PCR despite two preceding negative nasopharyngeal RT-PCR tests with importantly 13 of these 18 patients (72%) with two preceding negative nasopharyngeal RT-PCR tests having CT findings compatible with COVID-19 infection.^50^ This confirms the imperfection of single/multiple nasopharyngeal RT-PCR tests and that pulmonary changes compatible with COVID-19 infection in the context of a negative RT-PCR test(s) cannot be readily dismissed.

## Conclusion

Asymptomatic COVID-19 infection on FDG PET-CT is characterised by bilateral areas of GGO/consolidation that are associated with increased FDG uptake (>x2 liver SUV_mean_) and which can be identified with high reproducibility using the BSTI classification. These changes occur on the background of generalised inflammation within the mid-lower zones of the lungs, hilar and central mediastinal nodal stations, and spleen. This analysis will enable better preparedness for identification of asymptomatic COVID-19 infection on FDG PET-CT, prompting early confirmation RT-PCR testing, and minimising the risk of undetected infection to both the individual and society as a whole.

AVAILABILITY OF DATA AND MATERIALS: Anonymised data that supports these findings are available from the corresponding author upon reasonable request.
