# Supplementary material for: A case–control evaluation of pulmonary and extrapulmonary findings of incidental asymptomatic COVID-19 infection on FDG PET-CT
Source: Br J Radiol. 2021 Apr 29;95(1130):20211079. doi: 10.1259/bjr.20211079 (PMC8822569; doi:10.1259/bjr.20211079)
Supplement: Supplementary Material 1. [file bjr.20211079.suppl-02.docx]

**SUPPLEMENTARY INFORMATION**

**STATISTICAL ANALYSIS**

Inter-observer agreement for the BSTI classification based upon independent review by 2 reporters was assessed using the weighted κ method due to the ordinal nature of these categories: 0.81–1.00 = almost perfect agreement; 0.61-0.80 = substantial agreement; 0.41-0.60 = moderate agreement; 0.21-0.4 = fair agreement; 0.0-0.2 = slight agreement [33].

Association of SUV_max_ and TBR in GGO/consolidation by COVID-19 status (*confirmed* vs. *suspected* vs. *control* cases) and by BSTI classification (BSTI 1 vs. BSTI 2 vs. BSTI 3) was examined. Only the subgroup of *control* cases with visible areas of GGO/consolidation were eligible for this analysis. For each grouping (COVID-19 or BSTI classification), the non-parametric Kruskal-Wallis test was used to establish any group-wise differences, with subsequent pairwise tests between each level using the non-parametric Mann-Whitney U test. Four additional pairwise tests (Mann-Whitney U) were applied to aggregated groupings: comparing SUV_max_ GGO/consolidation in *confirmed* and *suspected* cases (COVID-19 group) vs. *control* cases and in BSTI 1 & 2 cases (BSTI 1 & 2 group) vs. BSTI 3 cases and comparing TBR GGO/consolidation in *confirmed* and *suspected* cases (COVID-19 group) vs. *control* cases and in BSTI 1 & 2 cases (BSTI 1 & 2 group) vs. BSTI 3 cases. To account for multiple comparisons, the 16 pairwise tests were corrected using the Benjamini-Hochberg method for estimating the false discovery rate (FDR). An FDR < 0.05 was the threshold for significant findings.

Receiver operating characteristic (ROC) curves were generated for the 2 aggregated groupings: SUV_max_ GOO consolidation COVID-19 group (*confirmed* and *suspected*) vs. *control* cases, and BSTI 1 & 2 group vs. BSTI 3 cases. The area under the curve (AUC) was calculated for each ROC [34] with the best SUV_max_ thresholds for group discrimination defined using Youden’s method (threshold cut-off providing the greatest sum of sensitivity and specificity) [35].

SUV metrics in the normal pulmonary parenchyma and extrapulmonary regions were compared in the aggregated COVID-19 group vs. *control* cases using the Mann Whitney U test; cases where malignant disease precluded placement of ROIs/VOIs for measurement of SUV metrics, were excluded from analysis. Again, the Benjamini-Hochberg method for estimating FDR was used to correct for multiple comparisons across these regions with FDR < 0.05 considered significant. All analyses were performed in R version 4.0.0 with the base and stats packages while ROC analyses were performed using the pROC package.

**SUPPLEMENTARY FIGURE LEGENDS**

**SUPPLEMENTARY FIGURE 1:** Scatter and box plots demonstrating differences in SUV metrics in generalised pulmonary parenchymal and extrapulmonary locations between the COVID-19 group and *control* cases (▲=CONFIRMED, ⚫=SUSPECTED, ■=CONTROL cases). Thick horizontal solid bar across the box shows the median, box height shows interquartile range (25-75^th^ percentiles) and whiskers show minimum and maximum values. * = p < 0.05, ** = p < 0.01, *** = p < 0.001.

**SUPPLEMENTARY TABLE LEGENDS**

**SUPPLEMENTARY TABLE 1:** Details of *suspected* cases of COVID-19 infection.

Abbreviations: BSTI = British Society of Thoracic Imaging, f/u = follow-up, RT-PCR = reverse transcriptase-polymerase chain reaction.

**SUPPLEMENTARY TABLE 2:** Comparison of generalised pulmonary and extrapulmonary SUV metrics between the COVID-19 group (*confirmed* and *suspected* cases)† and *control* cases††.

Abbreviations: * = statistically significant. RUZ = right upper zone, RMZ = right midzone, RLZ = right lower zone, LUZ = left upper zone, LMZ = left mid zone, LLZ = left lower zone, 10R = right hilar, 10L = left hilar, 2-4R = right upper and lower paratracheal, 2-4L, left upper and lower paratracheal, 5-6 = subaortic/aortopulmonary window and para-aortic, 7 = subcarinal, SUV_mean_ = mean standardised uptake value, SUV_max_ = maximum standardised uptake value, SD = standard deviation, FDR = false discovery rate. †a single *suspected* case was not assessed due to the disease involvement precluding analysis of ROIs and VOIs. ††three *control* cases were unable to be assessed to due to disease involvement precluding analysis of ROIs and VOIs. Nodal SUV_max_ was only measured if lymph nodes were visible on CT.
