# Supplementary material for: A case–control evaluation of pulmonary and extrapulmonary findings of incidental asymptomatic COVID-19 infection on FDG PET-CT
Source: Br J Radiol. 2021 Apr 29;95(1130):20211079. doi: 10.1259/bjr.20211079 (PMC8822569; doi:10.1259/bjr.20211079)
Supplement: Supplementary Table 1. [file bjr.20211079.suppl-03.docx]

| **Case** | **Scan indication** | **BSTI classification** | **RT-PCR status**  **(days after FDG PET-CT)** | **Management decision** | **6 month imaging follow-up** |
| --- | --- | --- | --- | --- | --- |
| 1 | Head & Neck cancer | 1 | Negative (1 day) | Self-isolation. Radiotherapy delayed by 1 week. | Resolution on 4 month f/u PET-CT. |
| 2 | Melanoma | 3 | None | Self-isolation. | Resolution on 2 month f/u CT thorax. |
| 4 | Head & Neck cancer | 2 | None | Self-isolation. Ultrasound FNA delayed by 2 weeks | Resolution on 2 month f/u CT thorax. |
| 6 | Melanoma | 2 | None | Self-isolation. | No further imaging. |
| 8 | Myeloma | 2 | None | Self-isolation. | Resolution on 5 month f/u chest radiograph. |
| 9 | Lymphoma | 1 | None | Self-isolation. | Resolution on 4 month f/u CT thorax. |
| 10 | Lymphoma | 3 | None | Self-isolation | No further imaging. |
| 11 | Melanoma | 1 | None | Self-isolation. Defer Nivolumab by 2 weeks | Resolution on 3 month f/u PET-CT. |
| 13 | Lymphoma | 2 | None | Self-isolation | Resolution on 1 month f/u PET-CT. |
| 14 | Endometrial cancer | 1 | Negative (1 day) | Self-isolation | Resolution on 2 month f/u PET-CT. |
| 15 | Lung cancer | 1 | None | Self-isolation | Resolution on 2 month f/u PET-CT. |
| 16 | Lymphoma | 3 | None | Self-isolation | No further imaging. |
| 17 | Oesophageal cancer | 3 | None | Self-isolation | No further imaging. |
| 18 | Unknown malignancy | 2 | Negative (1 day) | Self-isolation | No further imaging. |
| 20 | Melanoma | 1 | None | Self-isolation | Resolution on 5 month f/u PET-CT. |
