# Supplementary material for: A case–control evaluation of pulmonary and extrapulmonary findings of incidental asymptomatic COVID-19 infection on FDG PET-CT
Source: Br J Radiol. 2021 Apr 29;95(1130):20211079. doi: 10.1259/bjr.20211079 (PMC8822569; doi:10.1259/bjr.20211079)
Supplement: Supplementary Table 2. [file bjr.20211079.suppl-04.docx]

| **REGION** | **GROUP** | **Count** | **Minimum** | **Median** | **Maximum** | **Mean** | **SD** | **Uncorrected p-value** | **FDR** |
| --- | --- | --- | --- | --- | --- | --- | --- | --- | --- |
| **RUZ**  **SUV_mean_** | COVID-19 | 23 | 0.27 | 0.47 | 1.26 | 0.56 | 0.24 | 0.1767 | 0.2409 |
|  | CONTROL | 93 | 0.20 | 0.45 | 0.85 | 0.47 | 0.12 |  |  |
| **RMZ**  **SUV_mean_** | COVID-19 | 23 | 0.35 | 0.6 | 1.63 | 0.70 | 0.33 | 0.0033* | 0.0082* |
|  | CONTROL | 93 | 0.26 | 0.48 | 1.14 | 0.50 | 0.15 |  |  |
| **RLZ**  **SUV_mean_** | COVID-19 | 23 | 0.37 | 0.72 | 1.76 | 0.76 | 0.30 | 0.0001* | 0.0008* |
|  | CONTROL | 93 | 0.28 | 0.52 | 1.24 | 0.55 | 0.16 |  |  |
| **LUZ**  **SUV_mean_** | COVID-19 | 23 | 0.27 | 0.45 | 1.25 | 0.55 | 0.28 | 0.9200 | 0.9200 |
|  | CONTROL | 93 | 0.18 | 0.46 | 0.82 | 0.47 | 0.13 |  |  |
| **LMZ**  **SUV_mean_** | COVID-19 | 23 | 0.30 | 0.53 | 1.15 | 0.60 | 0.25 | 0.2376 | 0.2970 |
|  | CONTROL | 93 | 0.14 | 0.51 | 1.13 | 0.52 | 0.17 |  |  |
| **LLZ**  **SUV_mean_** | COVID-19 | 23 | 0.39 | 0.72 | 1.43 | 0.75 | 0.24 | 0.0012* | 0.0036* |
|  | CONTROL | 93 | 0.14 | 0.57 | 1.74 | 0.59 | 0.21 |  |  |
| **10R**  **SUV_max_** | COVID-19 | 22 | 1.32 | 2.82 | 6.57 | 3.17 | 1.31 | 0.0002* | 0.0008* |
|  | CONTROL | 92 | 1.15 | 2.03 | 7.97 | 2.21 | 0.88 |  |  |
| **10L**  **SUV_max_** | COVID-19 | 22 | 1.28 | 2.76 | 6.29 | 2.97 | 1.21 | 0.0002* | 0.0008* |
|  | CONTROL | 92 | 1.20 | 1.98 | 6.51 | 2.11 | 0.75 |  |  |
| **2-4R**  **SUV_max_** | COVID-19 | 14 | 2.20 | 3.36 | 10.7 | 4.01 | 2.26 | 0.0001* | 0.0008* |
|  | CONTROL | 43 | 1.10 | 2.00 | 9.83 | 2.34 | 1.45 |  |  |
| **2-4L**  **SUV_max_** | COVID-19 | 3 | 4.21 | 6.34 | 8.82 | 6.46 | 2.31 | 0.0250* | 0.0417* |
|  | CONTROL | 13 | 1.80 | 2.20 | 8.18 | 2.82 | 1.78 |  |  |
| **5-6**  **SUV_max_** | COVID-19 | 4 | 2.21 | 2.71 | 4.70 | 3.08 | 1.15 | 0.5042 | 0.5817 |
|  | CONTROL | 12 | 1.22 | 2.50 | 9.53 | 2.97 | 2.16 |  |  |
| **7**  **SUV_max_** | COVID-19 | 15 | 1.70 | 3.26 | 13.9 | 4.13 | 3.07 | 0.0158* | 0.0296* |
|  | CONTROL | 33 | 0.83 | 2.26 | 9.26 | 2.52 | 1.38 |  |  |
| **LIVER**  **SUV_mean_** | COVID-19 | 23 | 1.50 | 2.18 | 3.50 | 2.32 | 0.51 | 0.8816 | 0.9200 |
|  | CONTROL | 93 | 1.53 | 2.26 | 3.20 | 2.29 | 0.36 |  |  |
| **SPLEEN**  **SUV_mean_** | COVID-19 | 23 | 1.20 | 2.14 | 3.45 | 2.21 | 0.49 | 0.0106* | 0.0227* |
|  | CONTROL | 91 | 1.20 | 1.94 | 3.59 | 1.96 | 0.39 |  |  |
| **BONE MARROW**  **SUV_mean_** | COVID-19 | 23 | 1.13 | 2.20 | 3.73 | 2.20 | 0.61 | 0.0564 | 0.0846 |
|  | CONTROL | 93 | 1.04 | 1.86 | 4.00 | 1.96 | 0.57 |  |  |
